# Case report: A novel splice-site mutation of *MTX2* gene caused mandibuloacral dysplasia progeroid syndrome: the first report from China and literature review

**DOI:** 10.3389/fendo.2024.1345067

**Published:** 2024-03-13

**Authors:** Xiaohui Fu, Shuli Chen, Xiao Huang, Qinghua Lu, Yunfei Cui, Weinan Lin, Qin Yang

**Affiliations:** ^1^ Department of Inherited Metabolic Disorders, Shenzhen Children’s Hospital, Shenzhen, China; ^2^ Department of Respiratory Diseases, Shenzhen Children’s Hospital, Shenzhen, China

**Keywords:** *MTX2* gene, mandibular hypoplasia (MAD) syndrome, progeria syndrome, splice-site mutation, exon skipping

## Abstract

**Background:**

Mandibuloacral dysplasia (MAD) syndrome is a rare genetic disease. Several progeroid syndromes including mandibuloacral dysplasia type A (MADA), mandibuloacral dysplasia type B(MADB), Hutchinson-Gilford progeria (HGPS) and mandibular hypoplasia, deafness, and lipodystrophy syndrome (MDPL) have been reported previously. A novel MAD progeroid syndrome (MADaM) has recently been reported. So far, 7 cases of MADaM diagnosed with molecular diagnostics have been reported in worldwide. In the Chinese population, cases of MAD associated with the *MTX2* variant have never been reported.

**Methods:**

The clinical symptoms and the genetic analysis were identified and investigated in patients presented with the disease. In addition, we analyzed and compared 7 MADaM cases reported worldwide and summarized the progeroid syndromes reported in the Chinese population to date.

**Results:**

The present study reports a case of a novel homozygous mutation c.378 + 1G > A in the *MTX2* gene, which has not been previously reported in the literature. Patients present with early onset and severe symptoms and soon after birth are found to have growth retardation. In addition to the progeroid features, skeletal deformities, generalized lipodystrophy reported previously, and other multisystem involvement, e.g. hepatosplenic, renal, and cardiovascular system, this case was also reported to have combined hypogammaglobulinemia. She has since been admitted to the hospital several times for infections. Among 22 previously reported progeroid syndromes, 16/22 were MADA or HGPS caused by *LMNA* gene mutations, and the homozygous c.1579C > T (p.R527C) mutation may be a hot spot mutation for MAD in the Chinese population. MAD and HGPS mostly present in infancy with skin abnormalities or alopecia, MDPL mostly presents in school age with growth retardation as the first manifestation, and is often combined with an endocrine metabolism disorder after several decades.

**Conclusion:**

This is the first case of MAD syndrome caused by mutations in *MTX2* gene reported in the Chinese population. *MTX2* gene c.378 + 1G > A homozygous mutation has not been previously reported and the report of this patient expands the spectrum of *MTX2* mutations. In addition, we summarized the genotypes and clinical characteristics of patients with progeroid syndromes in China.

## 1 Introduction

Mandibuloacral dysplasia progeriod syndromes is a rare disabling autosomal recessive genetic disease first reported by Young et al. in 1971 (1). It is mainly characterized by severe skeletal deformities, abnormalities of skin, and lipodysplasia (2). It is estimated that there are about 400 children with progeria worldwide, it affects 1 in 4-8 million people approximately, irrespective of sex and race(Progeria 101 FAQ | The Progeria Research Foundation).Several types of MAD have been reported, of which the MAD associated with mutations in the *LMNA* gene is the first MAD syndrome with an identified gene (3). *LMNA* mainly causes Hutchinson-Gilford Progeria (HGPS) and type A MAD (MADA) (3, 4). HGPS is one of the progeria syndromes, Jonathan Hutchinson discovered it in 1886. The main features are progeroid face, short stature, skin abnormalities, lipodystrophy, skeletal abnormalities, etc. The differences between HGPS and MADA mainly lie in the molecular genetic pattern and the severity of the clinical phenotype, HGPS is usually characterized by earlier onset and more severe clinical symptoms than MADA, and in terms of molecular genetics, HGPS is predominantly autosomal dominant and is almost entirely caused by codon 608 mutation on chromosome 1 exon 11 of the *LMNA* gene, whereas MADA shows autosomal recessive inheritance (2). The other type of MAD, type B MAD (MADB), is caused by mutations in the gene encoding zinc metalloprotease *ZMPSTE24* (5). The clinical manifestations of MADB are similar to those of MADA. The main difference is that MADA is partial lipodystrophy, while MADB is generalized lipodystrophy and MADB presents with a more severe metabolic syndrome (6). Nestor-Guillermo Progeria Syndrome (NGPS) was first described and diagnosed in 2011 by Cabanillas R et al., with biallelic pathogenic variants in the *BANF1* gene (7). The product encoded by the *BANF1* gene is required for the reaggregation of lamin A during the remodeling of the nuclear envelope at the end of mitosis (7). Although the causative genes for several of the above syndromes are different, they are all thought to be associated with toxic accumulation of either prelamin A or lamin A/C.

In 2013, Weedon et al. identified a mandibular hypoplasia, deafness, and lipodystrophy syndrome (MDPL) caused by mutations in *POLD1* gene, which encodes the catalytic subunit (p125) of DNA polymerase δ(POLδ) (8). The catalytic subunit is responsible for synthesizing the lagging strand DNA during DNA replication with both 5′- to 3′-polymerase activity and 3′- to 5′-exonuclease activity (9). Polδ is involved in DNA replication and maintains genomic stability (9). The p125 mutation leads to reduced genomic stability, cellular senescence, and apoptosis, which may be the pathogenic mechanism of MDPL (9, 10). In contrast to other progeria syndromes, MDPL is characterized by early-onset hearing loss.

The novel MAD progeroid syndrome (MADaM: Mandibuloacral dysplasia associated to *MTX2*) reported in this study was first described in 2020 by Elouej et al., due to recessive mutations in *MTX2* encoding Metaxin-2 (MTX2), an outer mitochondrial membrane (OMM) protein (11). MADaM is clinically characterized by mandibuloacral dysplasia, generalized lipodystrophy, hypotonia, acro osteolysis, skin abnormalities, renal impairment, and cardiovascular system damage such as hypertension and left ventricular hypertrophy. To date, only 2 studies have reported 7 genetically definite diagnosed MADaM (11, 12). This study is the third, and the first MADaM from China.

## 2 Materials and methods

### 2.1 Patients and clinical investigations

A 2-year-4-month-old girl was admitted to Shenzhen Children’s Hospital in 2023, due to progeroid facial features, severe generalized lipodystrophy, hypotonia, skeletal malformations, recurrent liver enzyme elevations, eyelid edema and tachypnea. Multiple previous hospitalizations due to recurrent infections. Whole-exome sequencing was performed. A signed informed consent form was obtained from the proband’s parents.

### 2.2 Whole-exome sequencing

Whole blood (3 ml) was collected from the affected proband and her parents. DNA was isolated from peripheral blood with CWE9600 Automated Nucleic Acid Extraction System using CWE2100 Blood DNA Kit V2 (CWBiotech, China, CW2553). 750ng genomic DNA was fragmented into 200-300bp length using Scientz08-III Ultrasonic Homogenizer (SCIENTZ, China). The DNA fragments were then processed by end-repairing, A-tailing and adaptor ligation using KAPA Library Preparation Kit (Illumina, KR0453, v3.13), followed by an 8-cycle pre-capture polymerase chain reaction (PCR) amplification. Then, the amplified DNA sample was captured in the Agilent SureSelect XT2 Target Enrichment System (Agilent Technologies, Inc., USA). Captured DNA fragments were purified by Dynabeads MyOne Streptavidin T1 (Invitrogen, Thermo Fisher Scientific, USA) and amplified by 13 cycle post-capture PCR. The final products were purified by Agencourt AMPure XP (Beckman Coulter, Inc., USA) and quantitated with Life Invitrogen Qubit 3.0 by Qubit dsDNA HS Assay Kit (Invitrogen, Thermo Fisher Scientific, USA). Eventually, quantified DNA was sequenced in 150-bp paired-end modes on Illumina Novaseq 6000 platform (Illumina, Inc., USA) according to the standard manual.

The raw data produced on Novaseq platform were filtered and aligned against the human reference genome (hg19) using the BWA Aligner (http://bio-bwa.sourceforge.net/) after evaluated according to Illumina Sequence Control Software (SCS). The single-nucleotide variants (SNVs) were called using GATK software (Genome Analysis ToolKit) (www.broadinstitute.org/gatk). Variants were annotated using ANNOVAR (annovar.openbioinformatics.org/en/latest/). Effects of SNVs were predicted by SIFT, Polyphen-2, and MutationTaster programs. All variants were interpreted according to ACMG standards and categorized to be pathogenic, likely pathogenic, variants of unknown clinical significance (VUS), likely benign and benign.

## 3 Results

### 3.1 Case presentation

The proband, a 2-year-4-month-old girl, G3P2, was born to fourth-degree consanguineous parents at 38 weeks gestation after an uneventful pregnancy. The birth weight was 3350g and the height was in the normal range. Intellectual and motor development was within normal limits at the age of 1 year. After 1 year of age, language and motor development began to lag behind.

She had been suffering from lipodysplasia and recurrent diarrhea since about 5 months after birth. Elevated liver enzymes were noted at 8 months of age, at which point oral hepatoprotective drugs were started for more than a year. She was prone to recurrent infections and had eyelid edema that began when she was approximately 1 year old. A urine examination revealed massive proteinuria and microhematuria, and she had hypertension. Captopril and amlodipine were added to control her blood pressure, but the results were not satisfactory.

On physical examination, her weight was 6 kg (<3 p), height was 66.5 cm (<3 p), head circumference was 41.5 cm (<3 p) and blood pressure was 122/70mmHg. She had tachypnea at 42 breaths per minute, generalized lipodystrophy, poor skin elasticity, wrinkled skin, wide forehead, sparse hair and eyebrows, small jaw, full cheeks, patent fontanel, wet rales in both lungs, distend abdomen, generalized joint hypermobility, muscle hypotonia, and dystrophic nails.

**Figure 1:**
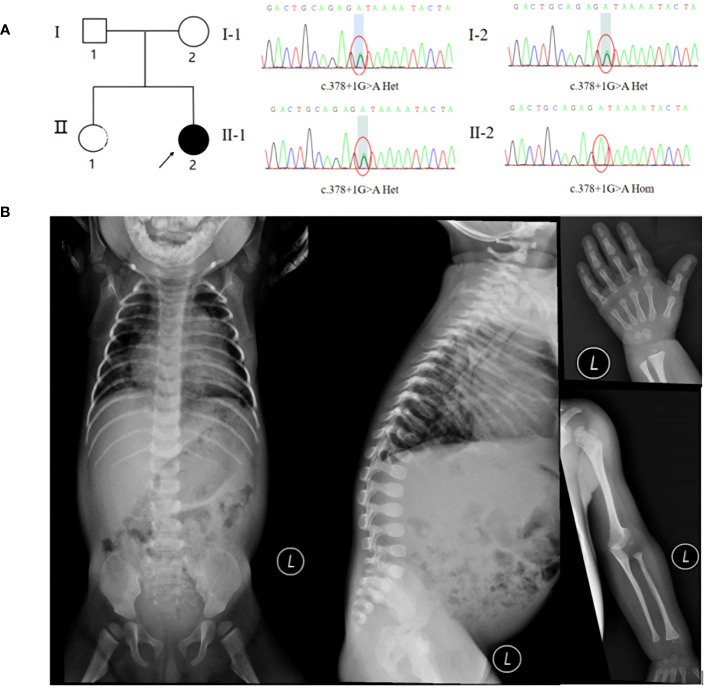
**(A)** Pedigree and Sanger sequencing of the MADaM family. **(B)** X-ray examination suggested pneumonia, mandibuloacral dysplasia, thoracolumbar kyphosis, developmental hip dislocation, gracile long bones of ribs, clavicles, and extremities, osteoporosis, osteolysis of the proximal radius and distal part of finger.

X-ray examination suggested pneumonia, mandibuloacral dysplasia, thoracolumbar kyphosis, developmental hip dislocation, gracile long bones of ribs, clavicles, and extremities, osteoporosis, osteolysis of the proximal radius and distal parts of both toes (**Figure 1B**). Previously elevated transaminases, ALT up to 471 IU/L, oral administration of liver protective drugs, this admission examination showed that the patient’s ALT was in the normal range, immune tests suggested IgG2.2g/l (3.2-11.5), cholesterol was 6.62mmol/L(0-5.18), urine examination suggested protein 3 +, occult blood 3 +, red blood cells 254 cells/UL (0-10).

### 3.2 Molecular diagnosis

**Figure 2:**
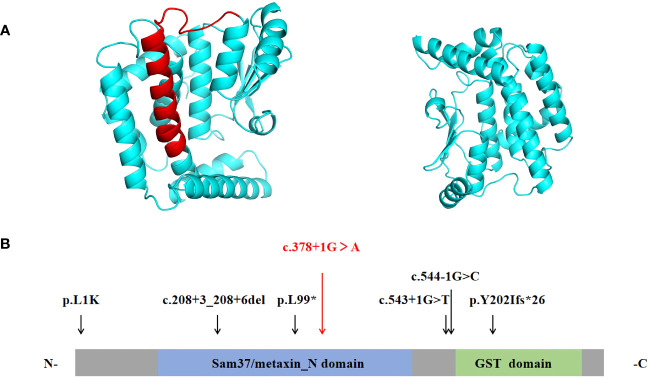
**(A)** The ribbon protein models of wild-type (left) and c.378 + 1G > A mutant forms (right) are displayed. The red section indicates the area where the mutant protein is lost. **(B)** Protein structure of *MTX2*. Previously reported mutations in the *MTX2* gene are indicated by black vertical arrows, and the mutation reported in this patient is shown in red. (N- N terminus; C-C terminus).

Coverage was 99.80%, the exon region and flanking splicing or intronic junctions of the *MTX2* gene were well covered, and the sequencing depth was >100×. Whole-exome sequencing revealed a homozygous *MTX2* gene mutation, NM_006554.5: c.378 + 1G > A, which had not been reported previously. The variant was inherited from his parents, and the mutation prediction retained the reading frame (**Figure 1A**). AlphaFold2 software was used to develop a suitable model to simulate the effect of the mutation region, the 3D protein modeling predicted that compared with wild type, the mutation would result in a truncated protein with an absence of the translated portion of exon 6 protein (**Figure 2A**). The mutation had not been reported in the normal population variant database (PM2), which was homozygous variation (PM3), but the deletion length accounted for more than 10% of the total protein length (PVS1). This variant can be rated as “likely pathogenic” (PVS1+PM3+PM2) according to ACMG guidelines.

## 4 Discussion

### 4.1 Mechanism

MADaM is caused by a mutation in the *MTX2* gene, which encodes a mitochondrial outer membrane protein known as Metaxin-2 (11, 13). Metaxin-2 (MTX2) interacts directly with Metaxin-1(MTX1), being part of the mitochondrial sorting and assembly machinery (SAM) responsible for the correct integration of β-barrel proteins into the outer mitochondrial membrane (14). Previous functional studies have shown that loss of MTX2 leads to decreased MTX1 protein levels, and causes mitochondrial network fragmentation, decreased oxidative phosphorylation, resistance to apoptosis triggering by TNF-α, increased senescence and autophagy, reduced proliferation, and abnormal nuclear morphology (11). These ultimately lead to severe features of progeroid in the patient.

### 4.2 Molecular mapping

*MTX2* located on chromosome 2q31.1, consists of 263 amino acids and contained two functional domains: Sam37/metaxin_N domain (41-162AA) and glutathione S-transferase (GST) domain (187-249AA) (https://www.ebi.ac.uk/interpro/protein/UniProt/O75431). The six previously reported *MTX2* variants in MADaM patients are summarized in **Figure 2B**, all variants were homozygous and from six consanguineous families (11, 12). There were three splicing mutations, two frameshift mutations, and one missense mutation. The c.378 + 1G > A mutation reported in this study is a splicing mutation, which is predicted to cause exon 6 skipping. Exon 6 of *MTX2* encodes 96 to 126 amino acids, and skipping of exon 6 is predicted to lose a portion of the Sam37/metaxin_N domain.

### 4.3 Clinical features

MADaM, like other types of MAD, also shown as autosomal recessive, and the patients reported so far are all homozygous mutations in the *MTX2* gene, which may be related to their consanguineous family history (11, 12). The clinical symptoms of MADA typically appeared at the age of 4-5 years, with most cases being partial lipodystrophy in the torso and limbs. The symptoms of MADB were more severe than those of MADA (6). All MADaM patients had a typical progeroid facial appearance, severe growth retardation, generalized lipodystrophy, clavicular hypoplasia, and distal osteolysis, indicating a severe progeroid form of MAD. In addition, 5/7 of the MADaM patients had hypotonia, which had not been reported in MAD patients previously. At present, MAD is considered to be a segmental form of progeria, mainly affecting bone, skin and adipose tissue, with the brain barely involved. So far, the reported cognitive development of MADA, MADB and MADaM is normal (**Table 1**) (11, 12, 15, 16).

**Table 1:** Clinical features of our patients and those 7 MADaM individuals reported in the literature.

	Our patient	MADaM (7 patients)
General information		
Gender	Femal	5 males/2 females
Age at onset (months)	5m	5m-24m,1NA
Weight/HeightHead	<3P/<3P	<3P/<3P
Progeroid facial appearance	+	(100%) 7+
Full cheeks	+	(100%) 7+
Sparse hair	+	(85%) 6+,1–
Hypotonia	+	(71%) 5+,2–
Generalized lipodystrophy	+	(100%) 7+
Atrophic skin, Pigmentation changes	+	(85%) 6+,1–
Skeletal abnormalities		
Delayed closure of cranial sutures	+	(85%) 6+,1–
Clavicular hypoplasia	+	(100%) 7+
Dysplastic femoral head	+	(85%) 6+,1–
Acroosteolysis of distal phalanges	+	(100%) 7+
Osteopenia	+	(85%) 6+,1–
Nail dystrophy	+	(100%) 7+
Cardiac involvement	–	(100%) 7+
Liver involvement	+	(43%) 3+,4–
Splenomegaly	–	(28%) 2+,5–
Renal involvement	+	(57%) 4+,3–
Hypertension	+	(85%) 6+,1–

(+) present; (−) absence. NA, not available.

Delayed closure of the cranial suture, clavicle hypoplasia and thin ribs due to osteolysis, acral osteolysis, joint contracture and nail dysplasia are common in almost all MAD cases (2). Dysplasia of the femoral head is rarely reported and has been reported in 6/7 of MADaM cases (11, 12). Occipital ossification defect is thought to be a unique feature of MADB (17), showing fragmented ossification on cranial imaging, which has not been found in MADaM to date. The ‘Beaten silver’ skull observed on the skull radiograph of the MADaM patient reported by Yeter Doğan et al. (12), may be related to increased intracranial pressure caused by cranial suture atresia, which was not found in our patient.

MAD patients tend to have thin wrinkled skin with pigmentary changes, which are also found in 6/7 of MADaM patients (11, 12). In addition, subcutaneous calcification has been found in patients with MADB (15), but not in patients with MADaM.

In contrast to other types of MAD, MADaM appears to be more likely to involve other organs at an early stage. Cardiac involvement was present in all 7 previously reported MADaM cases, including 4 cases with left ventricular hypertrophy and 3 others with dilated cardiomyopathy, mitral valve prolapse and patent foramen ovale, respectively (11, 12). Cardiac involvement has been identified in only 4 MAD patients so far (15, 18–20). With the exception of 1 case, a 4-year-old girl, who developed right heart failure due to recurrent infections, all but one occurred after the age of 20 years, and 2 after the age of 40 years. 6/7 of cardiac involvement in MADaM was left ventricular hypertrophy, probably related to severe hypertension in the patient.

Currently, there is no case report of MADA patients with renal involvement, and there are 6 cases of MADB (5, 6, 17, 18, 21), 2 of them eventually developed chronic renal failure in their second or third decade (5, 18). Most of MADaM had renal involvement (4/7) and hypertension (6/7) in the early stage, including 3 cases of focal segmental glomerulosclerosis and 1 case of IgM nephropathy (11, 12). The main manifestations of MADaM were proteinuria and microscopic hematuria. The patient reported in this study had massive proteinuria, hematuria and severe hypertension from the age of one year, and he gradually developed hyperlipidemia, despite treatment with enalapril with poor efficacy, similar to previous reports of MADB with chronic kidney disease.

Our patient presented with recurrent transaminase elevations before one year of age without hepatic dysfunction manifestations such as decreased albumin or increased bilirubin, and we excluded viral infections or other factors for hereditary liver disease. Hepatosplenic involvement is uncommon in MAD, but 3/7 of MADaM from our statistics had liver involvement (hepatomegaly or elevated transaminases) and 2/7 had splenomegaly (11, 12). Whether hepatosplenic involvement is a unique clinical feature of MADaM to distinguish it from other MAD requires more data in the future.

In this case, the patient also had a significant decrease in plasma IgG levels, which led to multiple hospitalizations due to infection. Low IgG levels have not been reported in previous patients with MADA, MADB, and MADaM. Clinical data have shown that the vast majority of children with nephropathy have obvious hypogammaglobulinemia, and the specific mechanism is not clear, and some studies suggest that it may be related to B-cell immunoglobulin class conversion dysfunction (22). Therefore, hypogammaglobulinemia in this patient may be a secondary manifestation of her nephropathy.

Few cases of MADaM have been reported so far, and its long-term prognosis is unknown. The oldest reported case of MADA is 56 years old, a Japanese woman who was paraplegic due to vertebral destruction at age 56 (23). A total of 5 patient deaths occurred, including 1 case of MADA and 4 cases of MADB (5, 15, 18, 24, 25). Two of the five patients died of respiratory failure in early stage (24, 25), and the rest died of kidney or heart failure in the second or third decade (5, 15, 18). In this case, the patient has been hospitalized for infection many times, and even developed respiratory failure, accompanied by complications such as elevated aminotransferases, nephropathy and hypertension. In addition to enalapril, dipyridamole and gamma globulin supplementation, we also tried to give the patient cocktail therapy, and the specific effect still needs long-term follow-up.

### 4.4 Characteristics of progeria syndrome in Chinese patients

So far, a total of 22 cases of progeria syndrome in the Chinese population have been reported, and their clinical features and genotypes are shown in **Table 2**. Among them, MADA or HGPS caused by *LMNA* gene mutation was the most common (72.7%), followed by MDPL caused by *POLD1* gene mutation (22.7%), and MADB was only 1 case, brain imaging examination showed typical failure of occipital ossification (35). The end-product of the *LMNA* gene lamin A is formed from the prelamin A (precursor protein of lamin A) through a series of shearing processing, which undergoes a series of processes such as farnesylation and hydrolysis by ZMPSTE24 enzyme to form mature lamin A (2). Lamins are part of the nuclear structural scaffold and play an important role in the structure and function of the nucleus. When *LMNA* or *ZMPSTE24* genes are mutated, they can lead to the toxic accumulation of prelamin A, leading to multiple types of premature aging laminopathies. The severity of the disease is mainly related to the degree of prelamin A accumulation (2).

Both MADA and HGPS are caused by mutations in the *LMNA* gene. The inheritance pattern of MADA is autosomal recessive, which is usually caused by homozygous or compound heterozygous mutations of *LMNA* (2). Previous reports have suggested that p.R527H is the most common mutation (20, 41). However, genetic analysis of Chinese progeria patients revealed that the *LMNA* gene p. R527C homozygous mutation is the most common mutation, and it is presumed that R527C is a hot spot mutation in Chinese MADA patients (**Table 2**). HGPS is thought to be autosomal dominant inherited disease and mostly caused by the p.G608G heterozygous mutation, which is known as classical HGPS (31). Individuals who exhibit the characteristic clinical features of HGPS, accompanied by other *LMNA* mutations, are often referred to as atypical HGPS(AHGPS) (33). However, since MADA has more overlap with HGPS clinical manifestations and is caused by mutations in the same gene, the clinical features cannot be easily distinguished (24). Plasilova et al. described the *LMNA* homozygous mutation p.K542N in four patients with an overlapping phenotype of MAD and early-onset progeria/HGPS, concluding that MAD and HGPS are a clinical spectrum disorder rather than separate disorders (24). HGPS clinical symptoms are generally characterized by an earlier onset and more severe clinical manifestations than MAD, with a mean life expectancy of 14.6 years, and the leading cause of death being stroke or myocardial obstruction (42). At present, three cases of HGPS caused by p.G608G heterozygous mutation have been reported in China, two of whom had cerebral infarction in childhood (31), which is a serious cardiovascular disease, suggesting the early involvement of cardiovascular system in HGPS.

**Table 2:** Clinical data review of Chinese progeria patients.

NO.	Form	Gender	Age at onset (symptom)	Genotype	Phenotype	Ref.
P1	MADA	Female	10m (mottled hyperpigmentation)	*LMNA*(hom) c.1579C>T (p. R527C)	mandibular hypoplasia, progeroid face, alopecia, short stature, skin abnormalities, B type lipodystrophy, skeletal abnormalities, swallowing difficulty, constipation	(26)
P2	MADA	Female	12m (mottled hyperpigmentation)	*LMNA*(hom) c.1579C>T (p. R527C)	mandibular hypoplasia, progeroid face, alopecia,short stature, skin abnormalities, A type lipodystrophy, skeletal abnormalities, swallowing difficulty, myogenic injury	(26)
P3	MADA	Male	8m (hypermyotonia)	*LMNA*(hom) c.1579C>T (p. R527C)	sparse hair, short stature, skin abnormalities, A type lipodystrophy, skeletal abnormalities, myogenic injury, hypertonia	(26)
P4	MADA	Male	1y (mottled hyperpigmentation)	*LMNA*(hom) c.1579C>T (p. R527C)	mandibular hypoplasia, progeroid face, sparse hair, skin abnormalities, B type lipodystrophy, skeletal abnormalities, bilateral internal carotid artery tortuous rotation	(27)
P5	MADA	Female	4m (Alopecia)	*LMNA* c.1579C>T (p.R527C), c.1583C>T (p. T528M)	mandibular hypoplasia, progeroid face, alopecia, skin abnormalities, A type lipodystrophy, skeletal abnormalities	(28)
P6	MADA	Male	6m (Alopecia)	*LMNA* c.1579C>T (p.R527C), c.1583C>T (p. T528M)	mandibular hypoplasia, progeroid face, alopecia, short stature, skin abnormalities, B type lipodystrophy, skeletal abnormalities	(28)
P7	MADA	Male	At birth (mottled hyperpigmentation)	*LMNA* c.1579C>T (p.R527C), c.1580G>A (p.R527H)	mandibular hypoplasia, progeroid face, sparse hair, skin abnormalities, lipodystrophy, skeletal abnormalities	(29)
P8	MADA	Female	1y (poor growth)	*LMNA* (hom) c.1580G>A (p.R527H)	mandibular hypoplasia, progeroid face, sparse hair, short stature, skin abnormalities, B type lipodystrophy, skeletal abnormalities, growth hormone deficiency, mild muscle hypertonia	(30)
P9	MADA	Male	NA	*LMNA* (hom) c.1580G>A (p.R527H)	mandibular hypoplasia, progeroid face, sparse hair, short stature, skin abnormalities, B type lipodystrophy	(30)
P10	HGPS	Male	1m (Scleroderma skin)	*LMNA* (hem) c.1824C > T (p.G608G)	mandibular hypoplasia, progeroid face, sparse hair, short stature, skin abnormalities, lipodystrophy, skeletal abnormalities, cerebral infarction(4y)	(31)
P11	HGPS	Male	20d(Sclerodermic skin)	*LMNA* (hem) c.1824C> T (p.G608G)	mandibular hypoplasia, progeroid face, alopecia, skin abnormalities, lipodystrophy, skeletal abnormalities, cerebral infarction(8y6m), bilateral hip dysplasia	(31)
P12	HGPS	Male	1m(Sclerodermic skin)	*LMNA* (hem) c.1824C> T (p.G608G)	mandibular hypoplasia, progeroid face, sparse hair, short stature, skin abnormalities, lipodystrophy, skeletal abnormalities	(32)
P13	AHGPS	Male	2m (Alopecia)	*LMNA* (hom) c.1579C>T (p. R527C)	mandibular hypoplasia, progeroid face, alopecia, short stature, skin abnormalities, B type lipodystrophy, skeletal abnormalities, myocardial ischaemia	(33)
P14	AHGPS	Female	4m (Alopecia)	*LMNA* (hom) c.1579C>T (p. R527C)	Alopecia, short stature, skin abnormalities, lipodystrophy, skeletal abnormalities	(33)
P15	AHGPS	Female	5m (Thickened skin of joints)	*LMNA*(hom) c.1579C>T (p. R527C)	mandibular hypoplasia, progeroid face, sparse hair, short stature, skin abnormalities, B type lipodystrophy, skeletal abnormalities, left ventricular hypertrophy, mild regurgitation of the tricuspid valve and pulmonary valve	(34)
P16	AHGPS	Male	6m (Alopecia)	*LMNA*(hom) c.1579C>T (p. R527C)	mandibular hypoplasia, progeroid face, alopecia, skin abnormalities	(34)
P17	MADB	Female	2y (poor growth)	*ZMPSTE24* c.743C>T (p.P248L), loss1(EXON:1-10)(all)	mandibular hypoplasia, progeroid face, sparse hair, short stature, skin abnormalities, B type lipodystrophy, skeletal abnormalities, occipital ossification defect, hyperuricemia, deformities of the external auricle	(35)
P18	MDPL	Female	3y (Poor growth)	*POLD1* (hem) c.1812_1814del (p.S605del)	mandibular hypoplasia, progeroid face, short stature, B type lipodystrophy, sensorineural hearing loss(10y),delayed menstrual periods	(36)
P19	MDPL	Female	7y (Poor growth)	*POLD1* (hem) c.1812_1814del (p.S605del)	mandibular hypoplasia, progeroid face, skin abnormalities, B type lipodystrophy, minimal breast development, fatty liver, conjunctival telangiectasia, sensorineural hearing loss(25y)	(37)
P20	MDPL	Female	3y (Poor growth)	*POLD1* (hem) c.1812_1814del (p.S605del)	mandibular hypoplasia, progeroid face, short stature, A type lipodystrophy, insulin resistance, fatty liver, sensorineural hearing loss(14y7m)	(38)
P21	MDPL	Female	4y (Poor growth)	*POLD1* (hem) c.3185A>G p.(Q 1062R)	mandibular hypoplasia, progeroid face, A type lipodystrophy, sensorineural hearing loss(6y)	(39)
P22	MDPL	Male	NA, diabetes presenting at 21y	*POLD1* (hem) c.3199G>A (p.E1067K)	mandibular hypoplasia, progeroid face, short stature, skin abnormalities, B type lipodystrophy, diabetes mellitus, hyperlipemia, carotid thickening with plaques, fatty liver, germinal aplasia	(40)
P23	MADaM	Female	5m (Poor growth)	*MTX2* (hom) c.378 + 1G > A	mandibular hypoplasia, progeroid face, sparse hair, short stature, skin abnormalities, B type lipodystrophy, skeletal abnormalities, elevated transaminases, renal involvement, hypertension, hyperlipemia, hypogammaglobulinemia	This study

Although previous reports suggested that clinical symptoms of MADA usually appear in early childhood (4-5 years) and MADB signs and symptoms can appear around the age of 2 years, our analysis of 22 progeria patients reported in China revealed that, except for five cases of MDPL mostly in preschool or school age where growth retardation was the predominant manifestation, most other progeria patients had clinical manifestations of progeria from infancy, mainly skin changes (8/17) and alopecia (6/17) (**Table 2**), and progressive skeletal and lipodystrophy with growth retardation, the main skeletal manifestation being progressive osteolysis of the extremities, which leads to hypoplasia of the mandible, terminal phalanges, clavicles, and ribs. Xu et al. performed a growth hormone provocation test in a Chinese MADA patient with the p.R527H homozygous mutation in the *LMNA* gene, which suggested partial growth hormone deficiency (30), and Sakka et al. reported that three MADA patients with the p.R527H homozygous mutation also had growth hormone deficiency, and they treated three patients with growth hormone for 18 months, but none of them had any effect (20), failure of response to trials of GH therapy in this patients could be a result of GH-IGF1 signaling disruption by pro-inflammatory cytokines, indeed IL-6 inappropriate secretion.

The early stage of MDPL is mainly characterized by growth retardation. *PODL1* gene c.1812_1814del(p.S605del) mutation has been reported as a hot spot mutation, which is also the most common mutation in China (3/5). One Japanese patient was examined with a growth hormone provocation test, which showed results suggestive of a normal range, sensorineural deafness was noted at ages 6-25 years, and effective treatment with hearing aids (43). In addition to this progeria, growth retardation and deafness, 4/5 patients had endocrine abnormalities, such as menstrual delay, gonadal dysgenesis, fatty liver, insulin resistance, diabetes and hyperlipidemia, which is similar to what has been reported abroad, and most MDPL patients had combined endocrine and metabolic abnormalities (**Table 2**).

## 5 Conclusion

We report the first case of MADaM syndrome caused by mutations in the *MTX2* gene in China, with some of the clinical features have not been previously reported. The clinical phenotypes of patients with progeroid syndromes often overlap, and early genetic testing is helpful for typing to provide clinical guidance for their treatment and prognosis follow-up.

## Data availability statement

The datasets presented in this article are not readily available because of ethical and privacy restrictions. Requests to access the datasets should be directed to the corresponding author.

## Ethics statement

The studies involving humans were approved by the Ethics Committee of Shenzhen Children’s Hospital. The studies were conducted in accordance with the local legislation and institutional requirements. Written informed consent for participation in this study was provided by the participants’ legal guardians/next of kin. Written informed consent was obtained from the individual(s), and minor(s)’ legal guardian/next of kin, for the publication of any potentially identifiable images or data included in this article.

## Author contributions

XHF: Software, Resources, Investigation, Funding acquisition, Data curation, Conceptualization, Writing – review & editing, Writing – original draft. SC: Writing – original draft, Methodology, Investigation. XH: Writing – original draft, Formal analysis, Data curation. QHL: Writing – original draft, Formal analysis, Data curation. YFC: Writing – original draft, Formal analysis, Data curation. WNL: Writing – original draft, Visualization. QY: Writing – review & editing, Supervision, Resources.
